# Facile H_2_PdCl_4_-induced photoreforming of insoluble PET waste for C1-C3 compound production

**DOI:** 10.3389/fchem.2023.1265556

**Published:** 2023-09-19

**Authors:** Dani Tong Li, He Yu, Ying Huang

**Affiliations:** ^1^ Stephen Perse Foundation, Cambridge, United Kingdom; ^2^ Laboratoire de Physique et d’Étude des Matériaux, ESPCI Paris, PSL Research University, Sorbonne Université, Centre national de la recherche scientifique, Paris, France; ^3^ Key Laboratory of Industrial Equipment Quality Big Data, No.5 Electronics Research Institute of Ministry of Industry and Information Technology (MIIT), Guangzhou, China

**Keywords:** photoreforming, plastic recycle, PET, energy regeneration, palladium

## Abstract

Plastic pollution has emerged as a pressing global concern, driven by the extensive production and consumption of plastic, resulting in over 8 billion tons of plastic waste generated to date. Conventional disposal methods have proven inadequate in effectively managing polymer waste, necessitating the exploration of novel techniques. Previous research has demonstrated the successful application of photoreforming (PR) in converting water-soluble oligomer fragments of plastics into valuable chemicals. However, an unresolved challenge remains in dealing with the insoluble oligomer fragments characterized by complex chemical structures and larger molecular sizes. In this study, we propose a facile approach that involves H_2_PdCl_4_-induced activation on PET substrate for PR of PET bottles. Remarkably, this method enables the production of C1-C3 compounds without the reliance on sacrificial reagents or photocatalysts. The significant findings of this study offer a practical solution to address the most formidable aspect of plastic PR, specifically targeting the insoluble oligomer fragments. Moreover, this research contributes to the advancement of effective strategies for the sustainable management of plastic waste.

## Introduction

Plastics have been vastly utilized in many fields attributing to their irreplaceable properties and low cost, but the excessive use of plastics consequently engenders an ever-growing environmental challenge (MacLeod et al., 2021; Bergmann et al., 2022; Liu et al., 2022; Sheridan et al., 2022). Particularly, 8 million tons of plastic waste are dumped into the oceans per year, and this continues to climb annually; it is predicted that marine plastic waste will outweigh the marine organisms by 2050 (MacArthur, 2017). Although plastic recycling technology has been rapidly developed, the recycling or utilization of plastics is still far from efficient and economical by traditional methods, especially for plastic of small size (microplastics, <5 mm). Plastic degradation is traditionally approached by thermal and stressful degradation methods, these protocols generally involve high temperatures (>500°C), high pressure (>10^3^ atm), low conversion efficiency (70%), and generate a wide product distribution (Songip et al., 1993; Songip et al., 1994). Alternatively, Garcia and Robertson (Garcia and Robertson, 2017) suggested that spent polyethylene terephthalate (PET) bottles (12% of totally produced plastic) were capable of being recast into bottles by shredding, melting, and remolding the polymer. However, the thermoplastic property of PET indicates low recovery and environmentally unfriendliness (Zhang et al., 2023). This PET waste challenge demands an additional approach to recycling polymer waste. The challenge is attributed to the complex chemical structures, extremely low solubility in water, and inefficient biodegradability.

An alternative solution is to employ plastic waste as feedstock for producing high-value chemicals, under mild and environmentally friendly conditions (Zhao et al., 2022; Dong et al., 2023). Previous works have focused on the photoreforming (PR) of plastic waste toward hydrogen generation via light-driven reactions (Uekert et al., 2018; Uheida et al., 2021), typically, under illumination (Uekert et al., 2018; Cao et al., 2022; Jiao et al., 2022; Ahrens et al., 2023), the semiconductive photocatalysts supply electrons and holes to conduct H_2_ evolution and plastic reformation, respectively. These works provided valuable insights for managing plastic waste at ambient temperature and pressure. To date, as the liquid supernatant containing high-value products attracts the attention of researchers, the study of PR concentrated on photocatalysis of the supernatant intermediates obtained from NaOH or KOH degreasing, mainly ethylene glycol and other soluble oligomer fragments. However, PR of the more challenging precipitants with larger molecular size and lower solubility remains unexplored and neglected. As the precipitant of plastic generally accounts for a high proportion (∼40%) and contains poisonous and environmentally unfriendly products, the research of supernatant illustrates the low transmission, low recovery efficiency, high cost, and low sustainability of PR research (Ikeda et al., 2013). There is an urgent need for PR plastic valorization to put forward a more economical, efficient, and practical idea.

Bond activation using transition metals (e.g., PdO_2_) on organic compounds is commonly efficient in manipulating chemical reactions and subsequently controlling the reaction rate or products (Li et al., 2021; Yang et al., 2021), suggesting a potential approach for transforming the precipitants of degreased plastic into valuable products (Bai et al., 2020; Dutta et al., 2022)^.^ Meanwhile, due to the degreasing reaction with NaOH, the long chains in the PET material are randomly broken, resulting in a large number of short chains of ethylene terephthalate and a series of by-products. In this way, NaOH promotes the interaction between catalytic and PET, thus enhancing the catalytic activity. However, the synergistic effect of NaOH with PdO_2_ and PET was not observed, leading us to consider the utilization of acidic Pd compounds (H_2_PdCl_4_) to facilitate the degradation of PET. Herein, we report a facile H_2_PdCl_4_-induced activation on PET substrate, for PR of PET bottles to produce C1-C3 compounds, without using any sacrificial reagent or photocatalysts. Simultaneously, the PR of PET bottles also contributes to hydrogen evolution under visible light illumination. After the reaction, over 99.8% of the Pd simple substance is successfully recycled and 59.1 wt% of the propiolic acid is produced. Propiolic acids have garnered significant research attention due to their diverse mechanisms, including nucleophilic and electrophilic properties, decarboxylative coupling and addition, and their broad applications, rendering them valuable chemical materials.

**FIGURE 1:**
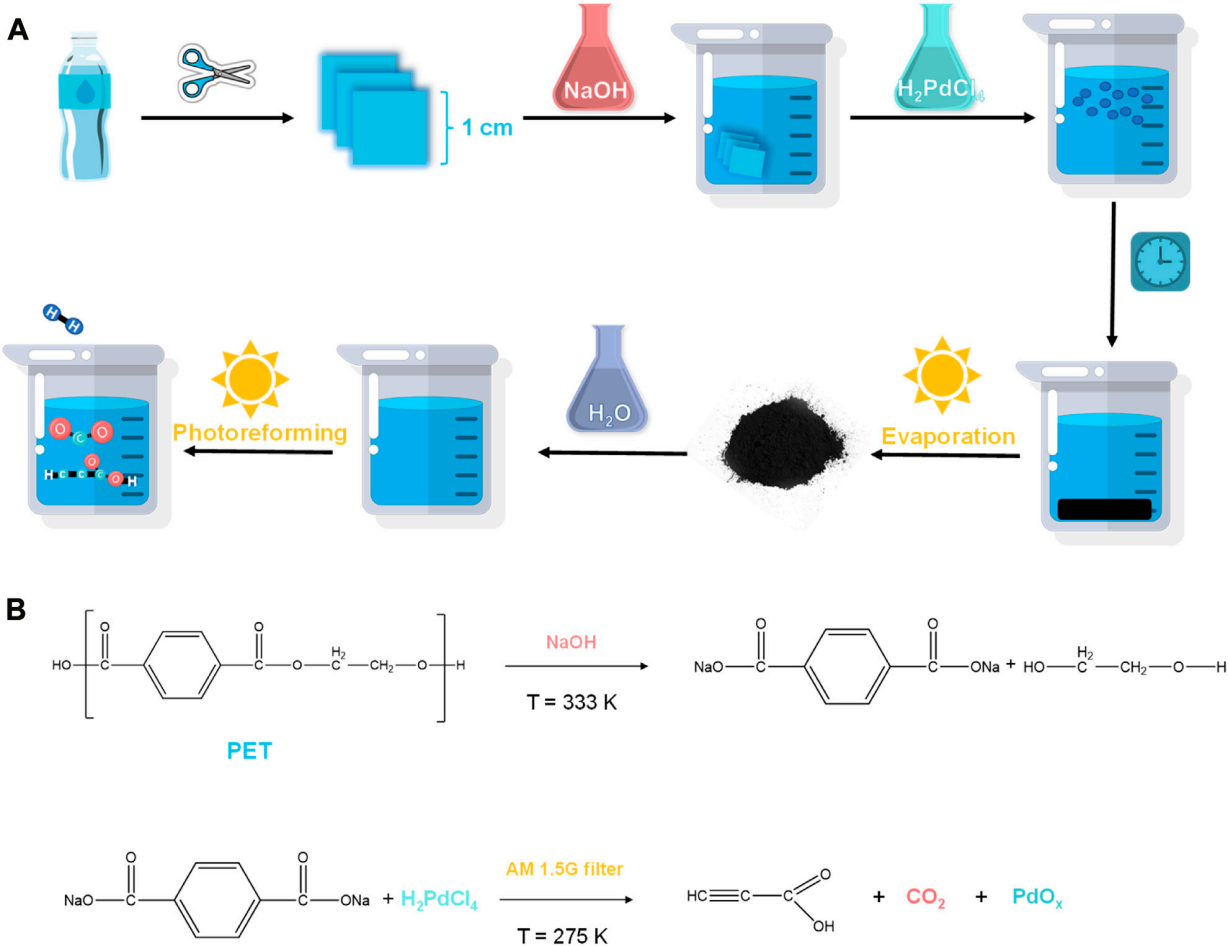
Photoreforming process. **(A)** From PET bottles to propiolic acid through the Pd ion activated photoreforming process. **(B)** The depolymerization reaction of PET to the monomer, and the photoreforming reaction of monomer.

This work has exceptional performances, including low cost, high sustainability, and high plastic degradation percentage, as well as highly valuable chemical materials production, suggesting a practical and innovative method for plastic degradation and propiolic acid production. Figure 1.

## Results and discussion

**FIGURE 2:**
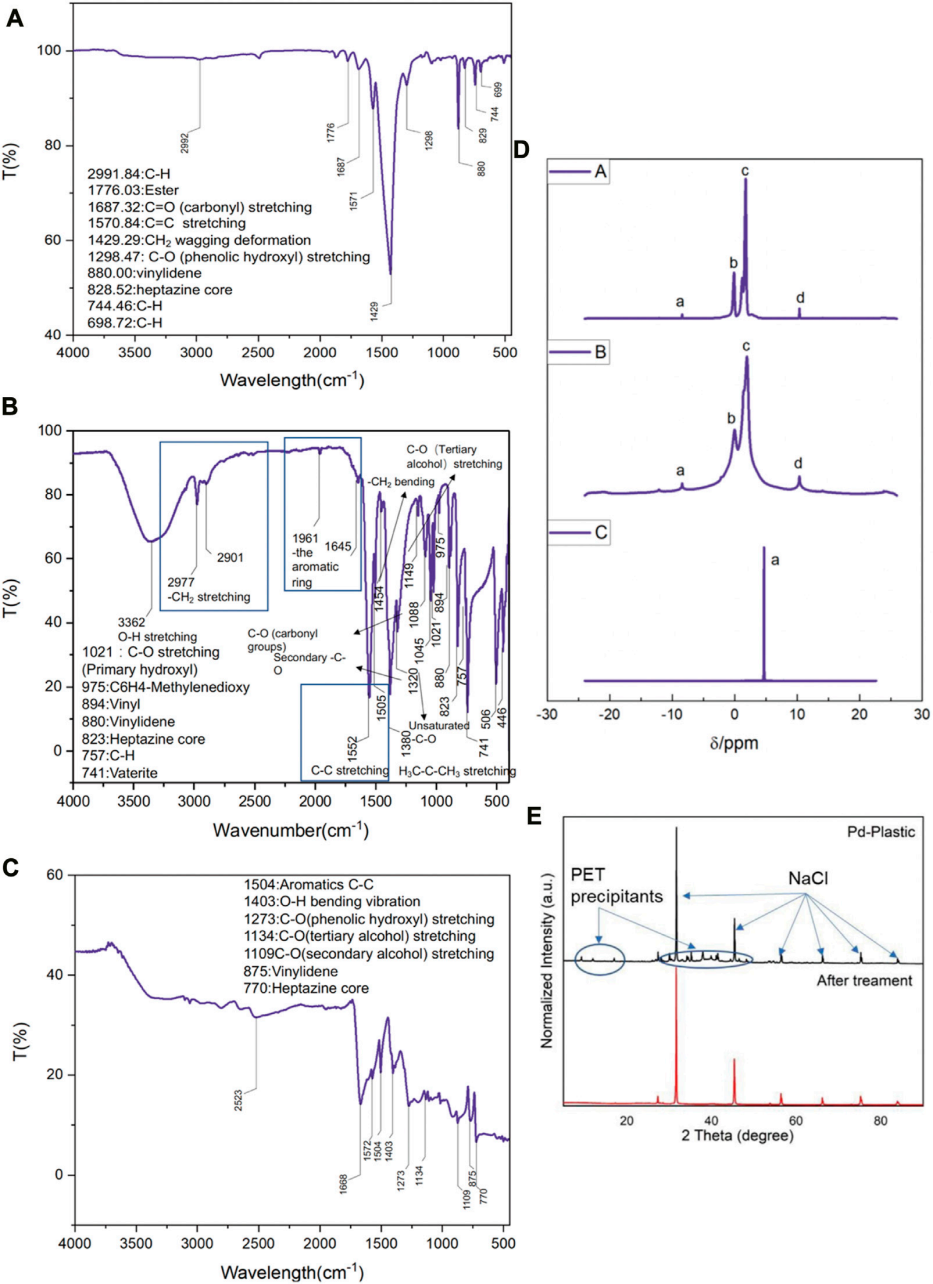
**(A)** FTIR of the sample A obtained from PET bottles after NaOH degreasing. **(B)** FTIR of the sample B obtained from A after H2PdCl4 treatment. **(C)** FTIR of the sample C obtained from B after 4 h illumination. **(D)** ^1^H NMR spectra of samples A, B, and C. **(E)** PXRD patterns of sample B before and after illumination treatment, NaCl (JCPDS No = 78-0751).

**FIGURE 3:**
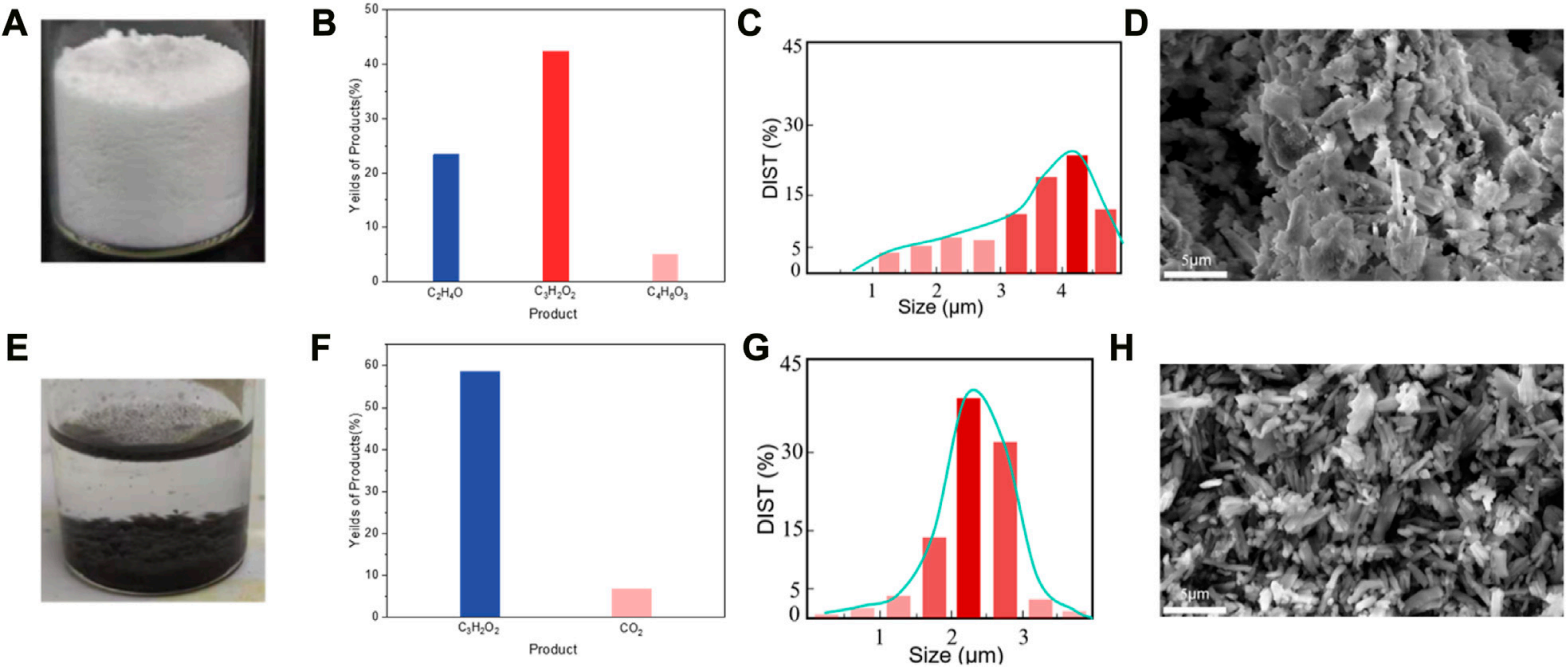
Pd activated degradation of the precipitants from degreased PET bottles. **(A)**, Digital photograph of the precipitants obtained from PET bottles after NaOH degreasing. **(B)**, The molar composition of precipitants was averaged from 5 individual tests and **(C)**, corresponding size distribution **(D)**, and SEM image. **(E)**, Digital photograph of precipitants treated by H_2_PdCl_4_. **(F)**, The molar composition of the liquid averaged from 5 individual tests and corresponding size distribution **(G)** and SEM image **(H)**.

In a typical experiment, 2.5 g of randomly collected waste PET bottles were cut into *ca*. 1 cm^2^ pieces, and then transferred in 0.5 L NaOH (10 M, aq.) under 60°C for 30 h for degreasing, followed which *ca*. 1 g of precipitants (Labeled as Sample A, white powder, Figure 3A) was collected. The precipitants obtained from the photoreforming process typically appear as a fine white powder, the texture of the precipitants can range from fine and powdery to slightly granular. It was confirmed to be carbon-hydrogen-oxygen compounds (Figure 3B) with complex chemical structures by combination tests of gas chromatography-mass spectrometry (GC-MS), Fourier transforms infrared spectroscopy (FTIR), and nuclear magnetic resonance (NMR; Figures 2A–D). The white precipitants presented flake morphology with an average size of *ca*. 3.5 μm as monitored by Zeta-sizer (Figure 3C) and scanning electron microscopy (SEM; Figure 3D). The addition of precipitants into H_2_PdCl_4_ aqueous solution (20 mL, 30 mM) triggered a violent release of hydrogen bubbles, accompanied by a rapid exothermic reaction owing to the pH neutralization between H_2_PdCl_4_ and the residual NaOH on precipitants, with a reaction time of approximately 20 s. The system turned to a colorless transparent liquid with black precipitants (Labeled as Sample B) with an average size of *ca*. 2 μm (Figures 3E, G, H) at pH 9.2, from which products were monitored in the liquid phase (Figure 3F). The obtained products exhibited smaller geometrical and molecular sizes relative to that before the treatment, although ^1^H NMR presented no obvious change in this stage (Figure 2D). As additional proof, powder X-ray diffraction (PXRD) also confirmed the trace of diffraction patterns from PET precipitants (Figure 2E).^11^ These results prove an efficient reaction between the large-size compounds in precipitants and the Pd-based acid, which drives the micro-plastic to nano-plastic in a very short time (*ca*. 20 s).

**FIGURE 4:**
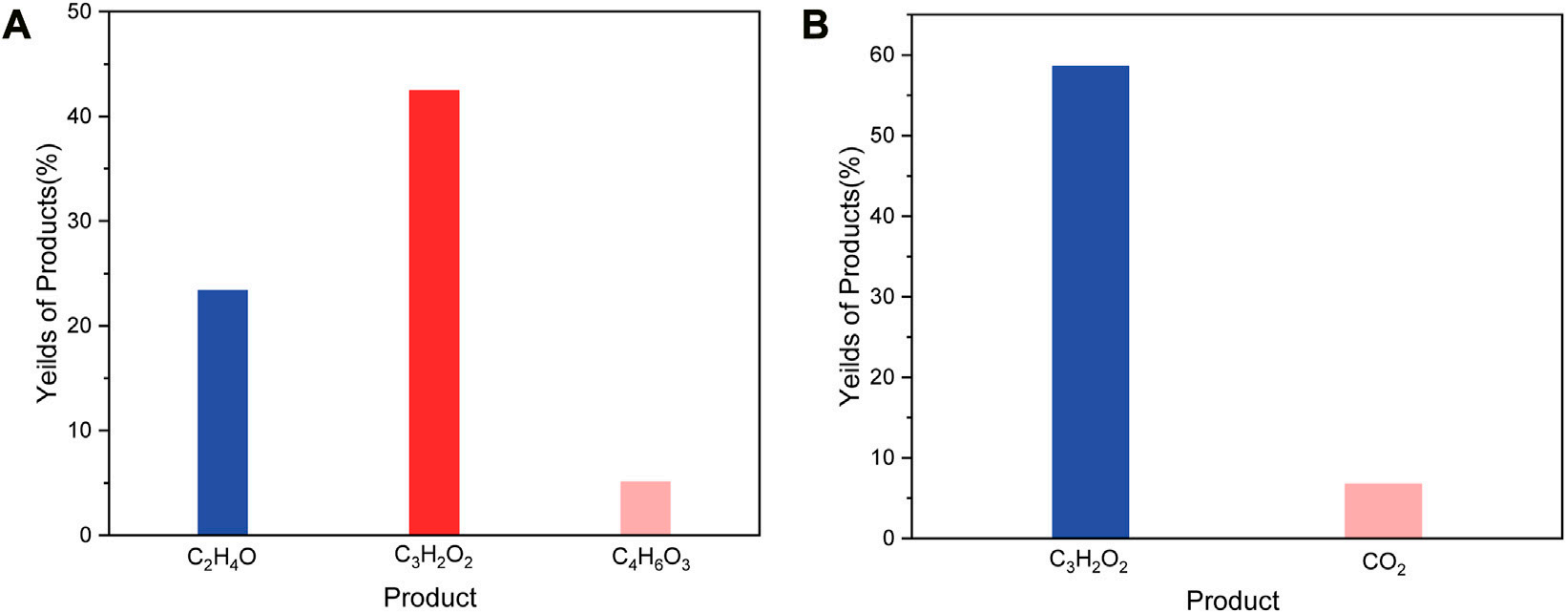
**(A)**, Products yield after 2 h Pd activated photoreforming over PET. **(B)**, Products yield after 4 h Pd activated photoreforming over PET.

**FIGURE 5:**
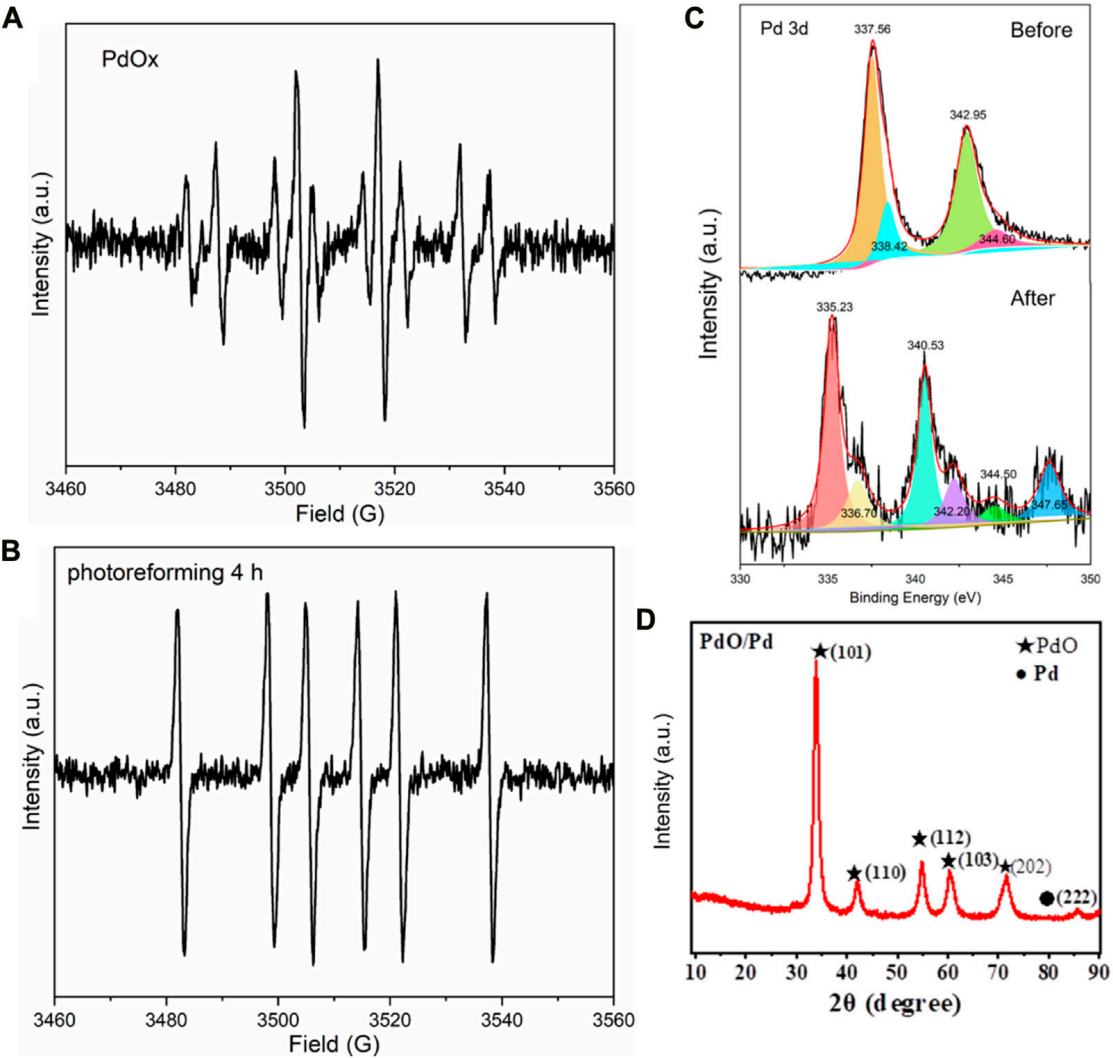
**(A,B)** EPR of sample B before and after illumination treatment. **(C)**, XPS of Pd before and after illumination treatment. **(D)** XRD of the obtained Pd species, JCPDS of PdO: 46-1043, JCPDS of Pd: 85-0713.

After H_2_PdCl_4_ treatment, the system was illuminated by simulated solar light (AM 1.5G filter) to drive the PR process. As shown in Figure 4A, the C2 to C4 compounds (Labeled as Sample C) were obtained as the main products after 2 h illumination (Figure 4B). In detail, the substance was degraded to predominant products consisting of 59.1 wt% of propiolic acid, 7.4 wt% carbon dioxide, and 5.1 wt% of hydrogen, relative to the weight of precipitants (1 g) from NaOH degreasing. Besides, after the PR reaction, the remaining weight of the precipitants was measured to be *ca*. 50 mg, indicating a conversion rate of ca. 95% according to the following calculation: $Conversion\;rate=\frac{M_{1}-M_{2}}{M_{1}}$ (Hu et al., 2022; Wang, et al., 2022), where M_1_ is the weight of reactants before PR, and M_2_ is the weight loss of reactants after PR. Furthermore, all the diffraction patterns from the PET precipitants were almost eliminated in this stage (Figure 2E), suggesting a successful decomposition of PET compounds. Further illumination conducted no product change and we found that the hydroxyl radical generation from PdO_x_ was terminated after 4 h PR reaction (Figures 5A, B) by electron paramagnetic resonance (EPR), meanwhile, the valence state of Pd exceeded the highest value recorded in the database. We proposed that the efficient PR was triggered at the expense of the valence state increase of Pd, until the PdO_x_ was insufficient to contribute hydroxyl radicals. After the termination of PR, the Pd was recycled in the formation of PdO_x_, and the residual Pd ion in the liquid was monitored to be ca. 0.17 wt% relative to the initially added Pd ion.

According to the comparison of the XPS patterns of Pd shown in Figure 5C, the evident changes consist of two kinds of peaks: 1) Sharp peaks: from the Pd(Ⅱ) (around 337 eV) and Pd 3d_3/2_ (around 343 eV) to the Pd(0) (around 335 eV) and Pd 3d_5/2_ (around 341 eV), the Pd compound participated in the oxidation reaction. After calculation, the quantity of Pd in sharp peaks accounts for 75.9%. 2) Intermediate peak: the Pd around 347 eV illustrates its higher valence than Pd^2+^, the Pd compound participated in the strong reduction reaction (the main reaction) (Ohtani et al., 1993; Brun et al., 1999). After calculation, the quantity of Pd in sharp peaks accounts for 22.5%. The changes in valence and chemical state suggest the combination of oxidation and reduction. The addition of H_2_PdCl_4_ to a photocatalytic reaction mixture of primary alcohols leads to the efficient production of the corresponding ethers and alkanes and the hemiacetal intermediate results in the strong reduction and oxidation of Pd particles (Litter, 1999; Guo et al., 2023).

As the product from PR of PET, the obtained propionic acids serve as not only the basic structural units for medical chemistry but also as the main component of the bioactive compounds and conducting polymers. For decades, research on propiolic acids has attracted a lot of attention according to their triggered mechanisms: 1) acids as nucleophiles; 2) electrophilic alkynes; 3) decarboxylative coupling; 4) decarboxylative addition; 5) electrophilic acids (Chen et al., 2020). The application of propiolic acids is abundant and attractive so it is defined as a valuable chemical material. Besides, after filtration and evaporation, the residual Pd ion in the liquid was monitored to be ca. 0.17 wt% relative to the initially added Pd ion, it thus represents the recovery of 99.8% from the PdO_x_ and Pd precipitates (Figure 5D). In summary, our research can not only provide an economical plastic degradation method but also produce valuable and necessary chemical material (Table 1).

**TABLE 1:** Recent research of plastic degradation.

Feedstock	Substrate	Method	Temperature	Additional condition	Product distribution	Conversion rate	References
PC	-	-	700 °C	Supercritical water/CO_2_ mixed environment	H_2_ (35.7%), CH_4_ (32.9%), CO_2_ (29.2%)	47.6% (PC to CH_4_ and CO_2_)	Zhao et al. (2022)
			29 atm				
PP	-	Thermochemical	600 °C	Spatial temperature gradient and temporal heating profile	CH_4_	36%	Dong et al. (2023)
PET	-		1 atm		C_2_H_4_, C_3_H_6_	43%	
PET	-	Binuclear catalyst	90 °C	biomimetic Zn‒Zn sites	ethylene glycol	<10%	Zhang et al. (2023)
			1 atm				
C-O bonds of epoxy resins and composites	-	Illumination	160 °C	Ru-catalyzed	p,p’-bisguaiacol F (57%), Me-BPA (50%)	-	Ahrens et al. (2023)
			1 atm				
PLA	Supernatant (C_3_H_6_O_3_)	Illumination	25 °C	CdS/CdO_x_ quantum dot photocatalyst	H_2_ (4.13%)	38.8%	Uekert et al. (2018)
PET	Supernatant (C_8_H_6_O_4_, C_2_H_6_O_2_)		1 atm		H_2_ (2.17%)	16.6%	
PUR	Supernatant (C_7_H_10_N_2_, C_3_H_8_O_2_)				H_2_ (5.15%)	22.5%	
PET	Supernatant (C_8_H_6_O_4_, C_2_H_6_O_2_)	Illumination	80 °C vacuum	MXene/Zn_x_Cd_1-x_S photocatalysts	glycolate, acetate, ethanol, H_2_	-	Cao et al. (2022)
PET	-	Illumination	25 °C	NiO/Fe_2_O_3_ photocatalyst	H_2_O, CO_2_	15.6% (PET conversion)	Jiao et al. (2022)
			1 atm				
PET	Precipitate	Illumination	25 °C	-	C_3_H_2_O_2_ (59.1 wt%), CO_2_ (7.4 wt%)	**>95%** (PET conversion)	**This work**
			1 atm				

^a^ PC, simple for Polycarbonate; PP, simple for Polypropylene; PLA, simple for Polylactic Acid; PUR, simple for Polyurethane.

## Conclusion

In summary, we have developed an innovative plastic recycling method. By degreasing and PR processing, we establish an innovative concept of plastic degradation and reformation. PR is so robust and environmentally friendly that this method is critically important for plastic degradation and valuable chemical materials production. Remarkably, the high production of propiolic acids (59.1 wt%) and high percentage recovery of Pd (99.8%) illustrates the high value of the product and low cost of reaction. With these sustainable and widely available properties, our method can become a strong competitor for landfilling plastic. Furthermore, given the widely available industrial production equipment, large-scale continuous production of our sustainable PR method for applications as an innovative method for plastic degradation can be expected in the future.

## Experimental procedures

In a typical experiment, 2.5 g PET flake (approximately 1 cm × 1 cm) was stirred in 10 M NaOH for 30 h at 60 °C followed by centrifugation to collect precipitants as substrate for subsequent experiments. The precipitate (1 g) was dissolved in 20 mL of deionized water, 20 mL of 30 mM H_2_PdCl_4,_ and 10 mL of 35 wt% H_2_O_2_ was added successively. The reactor was then illuminated by a 300 W Xe lamp with AM 1.5G filter, and the output of the light was adjusted to 100 mW·cm^−2^ (AM 1.5G filter). After filtration, washing, and evaporation, 99.8% of Pd was recycled as the black precipitate PdO_x_.

## Characterization techniques

Electron Paramagnetic Resonance (EPR) (BRUKER A300) was utilized to identify free radicals and hydroxyl groups. To examine hydroxyl groups and free radicals, simulated Sun irradiation was utilized.

For qualitative detection of molecular alterations in functional groups, FTIR spectroscopy (Thermo Fisher Nicolet IS 50/6700) was utilized. Using the ratio of the hydroxyl group, benzene ring, and ester group, the primary product of the reaction was estimated. Hydroxyl group is the ratio of absorbance peak regions at approximately 3,600 cm^−1^. The benzene ring is the ratio of absorbance peak regions at around 1600 cm^−1^. Ester group is the ratio of the regions beneath the absorbance maxima at around 1200 cm^−1^.

Under 300 °C, gas chromatography-mass spectrometry (GC-MS) (SHIMADZU QP2010ULTRA) was used to measure the spectral peak strength of ions and to separate and identify complicated components.

For analyzing the relative molecular mass of the products, a Gel Permeation Chromatograph (GPC) (WATERS ACQUITY APC) was utilized. And ^1^H nuclear magnetic resonance (NMR) spectra (BRUKER AVANCE III HD 400 MHz) were utilized to evaluate the interaction between spin nuclei and high magnetic fields, as well as analytical methods for detecting the molecular structure and chemical composition of samples.

The crystal structure of the materials was determined using an X-ray diffractometer (XRD) (Thermo Fisher Nexsa). Photoluminescence (PL) (Ocean Insight SR2) was utilized for the study of physical difficulties including electronic states, electronic transition processes, and electron-lattice interactions in solids. A constant 400 nm light source was used to detect the products under various situations.

## Data Availability

The original contributions presented in the study are included in the article/Supplementary Material, further inquiries can be directed to the corresponding author.
